# Effect of drying conditions on the preservation of selected bioactive compounds in Moringa oleifera aqueous extract: acetic acid, butyric acid, γ-aminobutyric acid, salicin, and glycine

**DOI:** 10.1186/s12870-025-07485-8

**Published:** 2025-10-22

**Authors:** Mehrdad Babarabie, Mahboobe Mohammadi, Amir Ghorbanzadeh, Sediqeh Afsharipour, Fatemeh Salari

**Affiliations:** 1 Department of Agriculture, Minab Higher Education Complex, University of Hormozgan, Bandar Abbas, 7916193145 Iran; 2 PhD in Horticultural Sciences, Faculty of Agriculture and Natural Resources, University of Hormozgan, Bandar Abbas, Iran; 3 Department of Horticulture, Faculty of Agricultural, Ferdowsi University of Mashhad, Mashhad, Iran; 4 Department of Horticultural Sciences, University of Hormozgan, Bandar Abbas, Iran

**Keywords:** Moringa oleifera, Drying, Secondary metabolites, Antioxidant activity, Glycine

## Abstract

**Background:**

Medicinal plants like *Moringa oleifera*, which possess high moisture content and microbial susceptibility, require prompt and efficient drying as a vital post-harvest step to ensure product stability and quality. This process is crucial for maintaining the plant’s sensory attributes and preserving secondary metabolites that are fundamental to its medicinal value and therapeutic effectiveness. The presence of specific functional small molecules and organic acids such as acetic acid, butyric acid, γ-aminobutyric acid (GABA), salicin, and glycine further amplifies moringa’s pharmacological potential. These compounds act as key signaling agents, regulating various physiological and biochemical pathways that support stress tolerance and health-promoting activities. Consequently, developing optimized drying strategies is essential to retain these beneficial bioactive components.

**Results:**

This study evaluated the effects of three drying methods—oven drying (50 °C for 24 h), shade drying (120 h), and sun drying (72 h) on the chemical composition and bioactivity of *Moringa oleifera* leaves. The experiment was designed as a randomized complete block with three replications. Key findings showed that sun and oven drying effectively preserved total phenolic compounds, flavonoids, salicin, and glycine. In contrast, shade drying was more effective at retaining antioxidant activity, as well as acetic acid, butyric acid, and gamma-aminobutyric acid (GABA). Notably, five bioactive compounds—acetic acid, butyric acid, GABA, salicin, and glycine were confirmed and further characterized in the aqueous extract of *Moringa oleifera* in this study.

**Conclusions:**

Overall, the findings of this study revealed that various drying methods have a significant impact on the preservation of key bioactive compounds in *Moringa oleifera*. Sun and oven drying were more effective in maintaining phenolic and flavonoid contents, whereas shade drying was better in preserving antioxidant activity and certain organic acids such as acetic and butyric acids. Additionally, specific compounds like salicin and glycine exhibited different responses to drying conditions; salicin reached its highest levels under sun drying, while glycine was maximized with sun drying and moderately retained during oven drying. These results emphasize the importance of selecting appropriate drying techniques based on the targeted bioactive compounds to optimize the medicinal value of herbal plants.

**Graphical Abstract:**

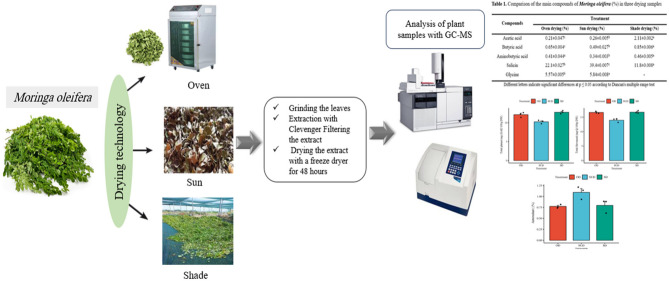

## Introduction

Moringa (*Moringa oleifera*) is a tropical tree native to India and Africa, primarily cultivated as a valuable leafy vegetable despite its timber being of relatively low quality. Its reputation as a “superfood” stems from its nutrient-rich leaves, which are abundant in essential minerals such as iron, calcium, and potassium, as well as vitamins A, C, D, and E [1]. In addition to these nutrients, moringa leaves are rich in reducing sugars and a wide array of bioactive phytochemicals, including alkaloids, terpenoids, flavonoids, anthraquinones, saponins, and tannins [2]. The leaf extract also contains significant levels of amino acids (proteins), omega-3 and omega-6 fatty acids, tocopherols (vitamin E), mineral salts (calcium and magnesium), phenolic acids (such as gallic and ferulic acid), and flavonoids like myricetin [3]. These compounds collectively contribute to the plant’s diverse pharmacological activities, which include antiviral [4], anti-inflammatory [5], hepatoprotective [6], and antidepressant properties [7]. Owing to these health benefits, dried moringa leaves are widely utilized as raw materials in the pharmaceutical, nutraceutical, confectionery, and animal feed industries [8].

Medicinal plants such as moringa have long been valued for their rich content of bioactive compounds that exert antiviral, antibacterial, anti-inflammatory, and antioxidant effects [9]. In this context, moringa’s therapeutic capacity is further supported by its content of several functional small molecules and organic acids, including acetic acid, butyric acid, GABA, salicin, and glycine. These compounds serve as critical signaling molecules involved in numerous physiological and biochemical processes that enhance the plant’s adaptability and health-promoting functions.

For instance, exogenous application of acetic acid has been shown to alleviate the detrimental effects of abiotic stresses such as drought, salinity, and heavy metal toxicity [10–12]. The underlying mechanisms include improved root and shoot development, enhanced photosynthetic efficiency and water-use performance, better stomatal regulation, increased chlorophyll content, and activation of antioxidant defense systems. Similarly, GABA, a non-protein amino acid, plays a central role in maintaining cellular carbon-nitrogen balance and facilitating nitrogen metabolism. GABA also promotes seed germination, accelerates plant development, and maintains redox homeostasis, thereby enhancing plant tolerance to abiotic stress and supporting higher crop productivity [13]. Salicin, a phenolic glycoside commonly found in willow bark, is another compound present in moringa. It is recognized for its anti-inflammatory and anti-irritant properties in dermatological applications. Advances in biomaterial science have led to the development of salicin-enriched collagen films that offer superior skin adherence, capitalizing on the therapeutic potential of this molecule [14]. Glycine, due to its high abundance, excellent water solubility, and low molecular weight, is often employed as a model compound in studies on organic nitrogen assimilation in plants. While research has shown its beneficial effects on root and leaf growth, its precise role in nitrogen signaling and metabolic modulation, particularly in crops like tea, remains to be fully elucidated [15].

Due to their high moisture content, medicinal plants are highly susceptible to spoilage, leading to considerable post-harvest losses. Drying is a widely adopted method to reduce such losses and extend the shelf life of these plants [16]. As a crucial step in the post-harvest processing of herbal materials, drying significantly influences the concentration and stability of volatile compounds and essential oils. Besides improving storage stability, drying also inhibits microbial growth and enzymatic activities, which are responsible for quality deterioration in herbal products [17]. However, the choice of drying technique and the specific conditions applied can greatly affect the medicinal properties of the plant material. Key quality parameters such as color, TPC, antioxidant activity, and essential oil composition are all influenced by the drying process. A comprehensive understanding of these impacts is essential for optimizing drying methods to ensure their suitability for applications in the food, pharmaceutical, and health-related industries [18].

Various drying techniques, such as air drying, oven drying, freeze drying, and sun drying, affect the physicochemical and functional attributes of medicinal plants differently [19]. Heat-based methods like air or oven drying can lead to degradation of essential bioactive compounds, including phenolics and volatile oils, which are critical for the plant’s therapeutic efficacy [20]. Nonetheless, drying not only extends shelf life but also facilitates the handling, packaging, and transportation of plant materials [21]. Modern drying technologies are categorized into three main groups: synthetic, natural, and hybrid methods. Synthetic approaches include conventional methods like hot air drying and advanced technologies such as microwave drying. Natural methods rely on sun or shade drying, which are cost-effective but often less controlled [22]. The primary objective in both scientific research and industrial applications is to enhance drying processes in a way that preserves the integrity of bioactive compounds, ensuring the therapeutic and nutritional quality of the final product [23]. Selecting the most appropriate drying method based on the nature of the plant’s active constituents is therefore critical, as it directly impacts the quantity and quality of the preserved bioactives [24].

Although numerous studies have been conducted on the bioactive composition of *Moringa oleifera* leaves, limited data are available on the effects of different drying methods on the retention of certain compounds in the aqueous extract of the plant. In the present study, the presence of five key bioactive compounds, acetic acid, butyric acid, GABA, salicin, and glycine, were analyzed in the aqueous extract of moringa leaves subjected to various drying techniques. Identifying and preserving these compounds can open new avenues for the medicinal and nutritional utilization of moringa and further emphasize the importance of selecting appropriate drying methods to safeguard its valuable bioactive constituents.

## Materials and methods

### Plant substance

**Fig. 1:**
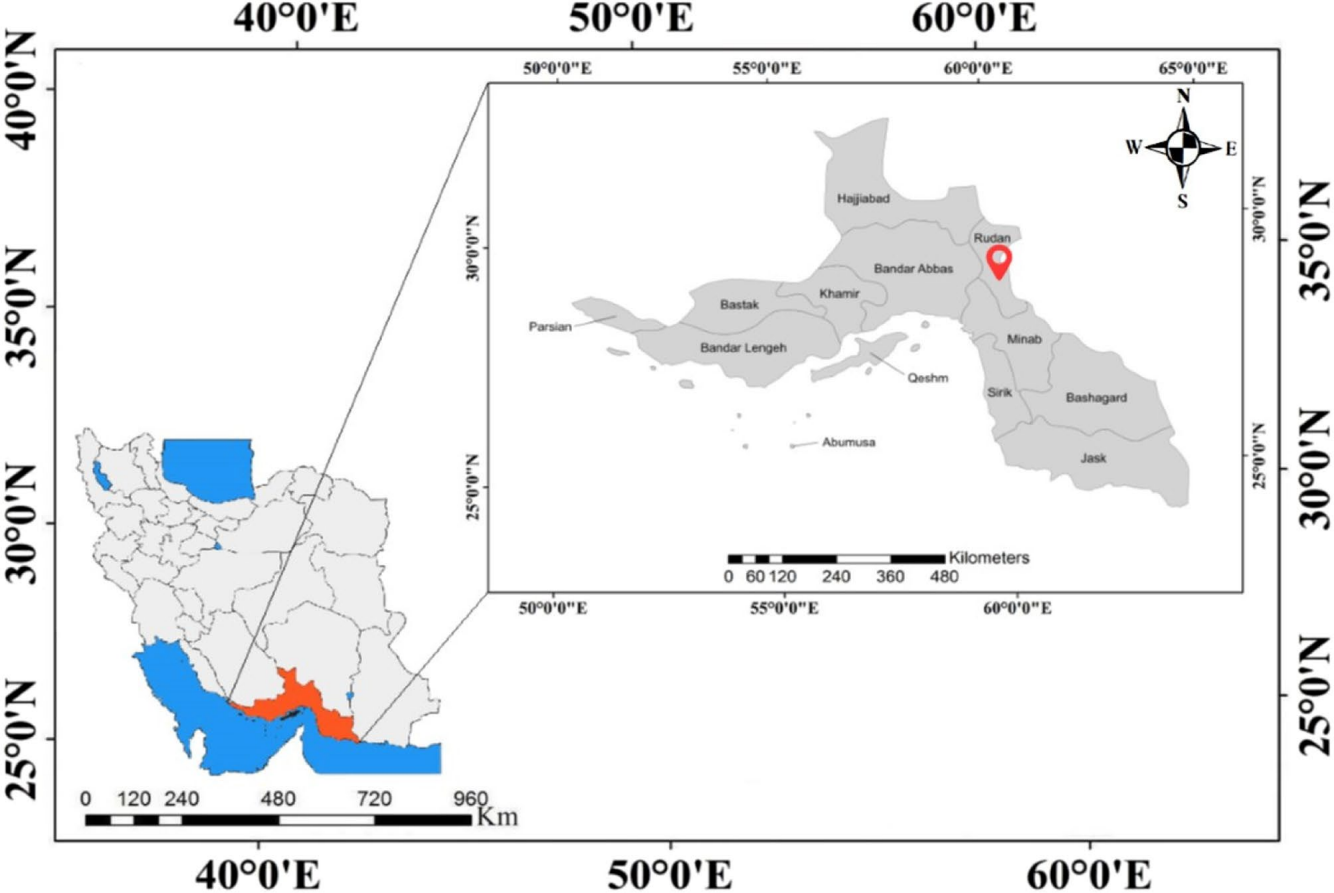
Moringa plant sample collection area

In June 2023, *Moringa oleifera* plant samples were collected from the Rodan region of Hormozgan Province, Iran, at an altitude of 190 m above sea level (geographical coordinates: 27°78’N, 57°19’E) (Fig. 1). The species was identified and confirmed by Dr. Mohammad Amin Soltanipoor, a faculty member at the Hormozgan Agricultural Research Center, Iran. The plant material came from a private garden in Rodan County, Hormozgan Province, belonging to one of the authors, Fatemeh Salari. Since her family cultivated and used the plant domestically for food and traditional medicinal purposes, no official permit was needed for collection. After collection, the samples were transported to Hormozgan University’s laboratory, where they were washed and disinfected using distilled water. Three biological replicates were prepared for all sampling procedures to ensure accuracy and reproducibility.

## Methods and tools for drying

The drying of samples was conducted on a wet weight basis until the moisture content reached an anticipated 10%. The mass loss of the samples during the drying process was carefully documented using an analytical balance. Three different drying methods were employed to dehydrate the leaves: Oven Drying (OD): The leaves were placed in an oven (Martin Christ, Germany; model Alpha 1–2 LDplus) at 50 °C for 24 h. Sun Drying (SUD): The leaves were spread on a blank sheet of paper placed on the open ground and exposed to direct sunlight for 72 h. Shade Drying (SD): The leaves were arranged on drying frames inside a dimly lit, well-ventilated chamber at 25 ± 2 °C for 120 h, avoiding direct exposure to sunlight [25]. Each drying process was repeated three times to ensure accuracy, and three biological replicates were considered for each method. After drying, the leaves were ground into a fine powder using an electric grinder.

### Drying time

The amount of time needed to achieve the final weight, or 10% of the initial moisture content, was used to record the drying process length for each approach.

### Pace of drying

By measuring the amount of mass loss throughout the drying process, the drying rate was calculated and then expressed in grams per hour.

### Extraction

Dried Moringa leaves were extracted using a Clevenger apparatus for 4 h at 100 °C and with a leaf-to-distilled water ratio of 1:10. After filtering the extract, the supernatant was dried for 48 h using a freeze dryer.

### Total phenolic content (TPC) and total flavonoid content (TFC)

To make the methanolic extract, 500 mg of Moringa extract was combined with 1.5 mL of 85% methanol, and then centrifuged for 10 min at 13,000 rpm following a 24-hour rest period.

The technique described in the reference [26] was employed, with minor modifications, to quantify the extract’s total phenolic content (TPC), total flavonoid content (TFC), and antioxidant activity. After combining 150 µL of methanolic extract from each sample with 750 µL of Folin-Ciocalteu reagent, the samples were allowed to rest for five minutes. 600 µL of 7% Na_2_CO_3_ was then added. Following a one and a one-and-a-half-hour dark period in a shaker, a UV-visible spectrophotometer (CECIL-2501, England) was used to detect the absorbance at a wavelength of 750 nm. Gallic acid equivalent (mg GAE/100 g DW) was used to express the results.

The aluminum chloride colorimetric technique was used to measure TFC concentrations [27]. In short, 600 µL of 85% methanol was combined with 200 µL of the methanolic extract. The samples were then supplemented with 40 µL of 10% aluminum chloride, 40 µL of 1 M potassium acetate, and 1120 µL of distilled water. The samples were shaken in the dark for 30 min, and their absorbance at 415 nm was measured using a UV-VIS spectrophotometer (CECIL-2501, UK). Using quercetin values as a reference, a calibration curve was created. The data were displayed as (mg Q/100 g DW).

### Antioxidant activity

The 2,2-diphenyl-1-picrylhydrazyl (DPPH) radical scavenging test was conducted using the methodology described in [28]. Consequently, 1170 µL of DPPH and 30 µL of methanolic extract were combined, and each sample was agitated for 10 s. A UV-VIS spectrophotometer was used to test the mixture’s absorbance at a wavelength of 517 nm following 30 min in the dark. The following formula was used to determine the DPPH radical scavenging activity:

$$\:AA=\frac{{A}_{c}-{A}_{s}}{{A}_{c}}$$

Where AA is the antioxidant activity (%), Ac is the absorbance of the control, and As is the absorbance of the sample. A refers to absorbance.

### GC/MS analysis

Gas chromatography–mass spectrometry (GC–MS) analysis of the Moringa extract was conducted using an Agilent Technologies, USA, model 7890 A. The instrument was equipped with a flame ionization detector (FID) and a VF-624ms column. The oven temperature was initially set at 60 °C for 2 min, followed by a ramp of 3 °C/min until reaching 150 °C. Subsequently, the temperature was increased at a rate of 5 °C/min to a final temperature of 270 °C, which was held for 15 min. Helium was used as the carrier gas at a constant flow rate of 1.1 mL/min. The injector and detector temperatures were both set at 300 °C, with a split ratio of 1:50. Electron ionization (EI) was employed with an ionization energy of 70 eV. The identification of volatile compounds was performed by comparing the obtained mass spectra and retention indices with those reported by Adams [29], using the Wiley library as a reference. The percentage values of each compound reported in Table 1 represent the relative peak area (%) of each identified compound. These were calculated based on the ratio of the individual compound’s peak area to the total peak area of all detected compounds in the chromatogram, multiplied by 100. Peak areas rather than peak heights were used to ensure accuracy and reproducibility. All measurements were performed in triplicate for reliability.

### Statistical analysis

This study used a randomized complete block design (RCBD) with a factorial experiment. At a significance level of α = 0.05, mean values from different treatment combinations were assessed using least significant difference (LSD) testing. SAS software version 9.4 was used for the statistical analysis.

## Results

### Investigating the effect of drying methods on the total phenol content in the Moringa plant

**Fig. 2:**
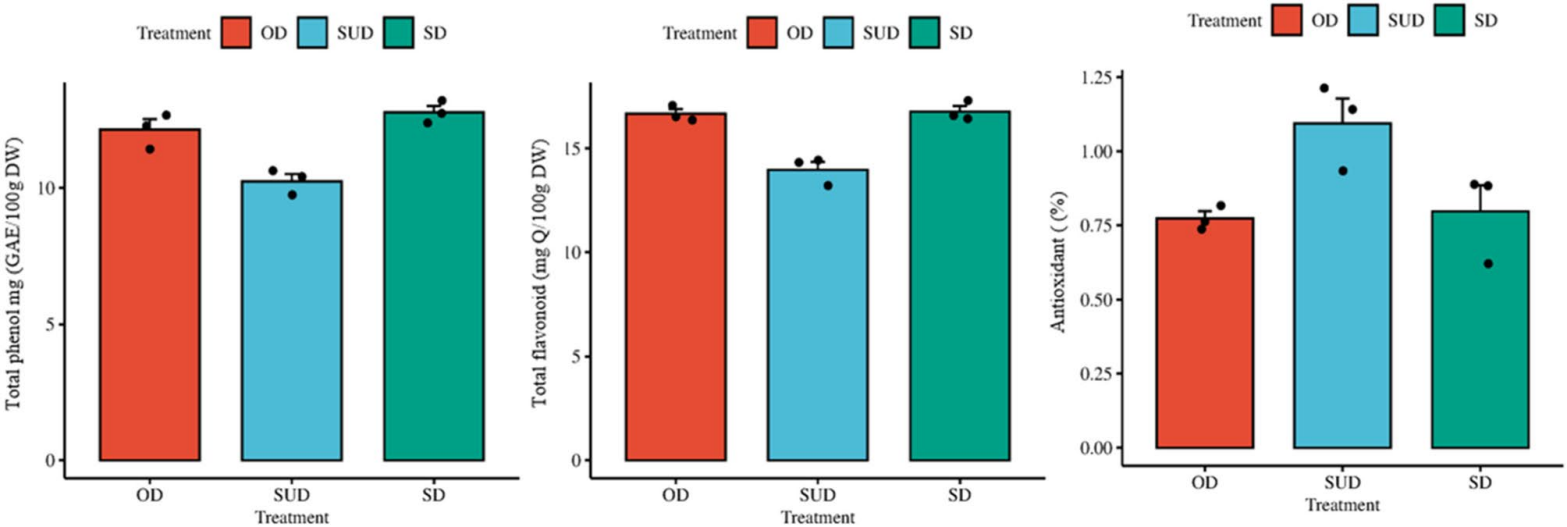
The effect of treatments (Oven drying: OD, Sun drying: SUD, Shade drying: SD) on total phenols, b) flavonoid capacity, and c) antioxidant activity of Moringa oleifera. The error bars show the standard errors (SE) of the means, while the data show the mean values of *n* = 3. The LSD test was used for statistical analysis at the *p* ≤ 0.05 level

Phenols are important antioxidant compounds found in medicinal plants. In investigating the effect of drying methods on the TPC of Moringa leaves, the results showed that oven drying (OD) and shade drying (SD) significantly maintained higher levels of TPC than sun drying (SUD). The highest phenol content was observed in the OD treatment, and the lowest in the SUD treatment. Based on the LSD test, the difference between OD and SD was not significant, but both were statistically significant (*p* ≤ 0.05) compared to SUD (Fig. 2).

### Investigating how drying techniques affect the Moringa plant’s overall flavonoid content

Regarding the amount of flavonoids, shade drying (SD) and oven drying (OD) treatments showed higher values ​​compared to sun drying (SUD) treatment. The highest amount of flavonoids was observed in SD treatment, which was in the same statistical group as OD and was significantly higher than SUD treatment. This result indicates that flavonoids are better preserved under shade and oven conditions.

### Examining how drying techniques affect the Moringa plant’s overall antioxidant activity

When assessing the quality of medicinal plants, antioxidant activity—which shows a substance’s capacity to scavenge free radicals and prevent oxidative damage—is regarded as a crucial metric. The findings indicate that the drying process has a major impact on Moringa’s overall antioxidant activity. In the evaluation of antioxidant activity, the sun-drying (SUD) treatment showed the highest antioxidant activity, which was significantly higher than the OD and SD treatments. In contrast, the lowest antioxidant activity was observed in the SD treatment. These results indicate that direct sunlight can play a positive role in maintaining or increasing antioxidant compounds, while shade drying may lead to a decrease in this activity.

### Examining how drying techniques affect active ingredients

In this study, the effects of three drying techniques, oven, sun, and shade drying, on the concentration of several active chemicals in a plant sample were examined. The findings indicate that the amount of acetic acid, butyric acid, GABA, salicin, glycine, TPC, TFC, and antioxidant activity is considerably impacted by the drying process.

### Acetic acid

The findings demonstrated that the drying process had a major impact on the amount of acetic acid. Shade drying resulted in the highest acetic acid content (2.11%). In contrast, sun and oven drying showed lower levels of acetic acid. These findings suggest that environmental factors present in the shade, such as low light and controlled humidity, can be effective in maintaining and increasing the acetic acid content in the plant.

**Table 1:** Comparison of the main compounds of *Moringa Oleifera* (%) in three drying samples

Compounds	Treatment		
	Oven drying (%)	Sun drying (%)	Shade drying (%)
Acetic acid	0.21 ± 0.047^c^	0.26 ± 0.005^b^	2.11 ± 0.002^a^
Butyric acid	0.65 ± 0.004^c^	0.49 ± 0.027^b^	0.85 ± 0.006^a^
Aminobutyric acid	0.41 ± 0.044^a^	0.34 ± 0.003^b^	0.46 ± 0.005^a^
Salicin	22.1 ± 0.027^b^	39.4 ± 0.007^c^	11.8 ± 0.008^a^
Glycine	5.57 ± 0.005^b^	5.84 ± 0.008^a^	-

Different letters indicate significant differences at *p* ≤ 0.05 according to Duncan’s multiple range test

### Butyric acid

The results obtained show that the drying method significantly affects the amount of butyric acid. So, drying in the shade resulted in the highest amount of butyric acid (0.85%). In contrast, drying in the sun showed the lowest amount of butyric acid (0.49%). Drying in the oven also showed a moderate amount of butyric acid (0.65%). To preserve butyric acid in medicinal plants, drying in the shade with low light and controlled humidity is the best method, as direct sunlight and inappropriate temperatures cause the compound to decompose.

### Aminobutyric acid

The findings indicate that the amount of butyric acid is significantly impacted by the drying process. Drying in the shade showed the highest content of butyric acid (0.46%) while drying in the sun yielded the lowest content (0.34%). So, oven drying also showed a moderate content of butyric acid (0.41%). Drying in the shade with low light and controlled humidity is the best way to preserve the amino acid butyric acid in medicinal plants. Direct sunlight and improper temperatures cause the compound to decompose.

### Salicin

The results obtained show that the drying method significantly affects the amount of salicin. Sun drying resulted in the highest amount of salicin (39.4%), indicating the positive effect of direct sunlight on the preservation or increase of this compound. In contrast, shade drying showed the lowest amount of salicin (11.8%). Oven drying also showed a moderate amount of salicin (22.1%). Thus, the quantity of active ingredients, like salicin, in medicinal plants can be greatly impacted by selecting the right drying technique. In the case of salicin, sun drying is recommended as the best method to preserve and increase this compound.

### Glycine

Glycine, an amino acid with various physiological functions, is a compound of interest in herbal medicine. The findings demonstrated that the drying process had a major impact on the glycine levels. Specifically, sun-drying resulted in the highest glycine content (5.84%), while oven-drying yielded a moderate level (5.57%). This finding hypothetically suggests that exposure to direct sunlight could be associated with enhanced synthesis or preservation of glycine in the plant material. The moderate level of glycine observed in oven-dried samples could be attributed to the controlled heat, which might have partially preserved the compound. Therefore, the choice of drying method can significantly affect the glycine content of medicinal plants. Therefore, sun drying may be the most effective method to maximize glycine levels.

## Discussion

Alkaloids, flavonoids, terpenoids, saponins, and phenolic chemicals are examples of secondary metabolites found in medicinal plants that have anti-inflammatory, antioxidant, anticancer, and antibacterial qualities [29, 30]. Herbal remedies, dietary supplements, and cosmetics are frequently made with these substances [31]. Additionally, researchers have placed a lot of emphasis on medicinal plants as environmentally safe and sustainable substitutes for synthetic substances [32]. The quality and effectiveness of medicinal plant compounds are crucially dependent on appropriate post-harvest processing, particularly drying techniques, given the growing demand for natural products and herbal alternatives in the treatment of illnesses [33]. One of the most crucial post-harvest procedures for medicinal plants is drying, which has a direct impact on the plants’ quality, shelf life, and bioactive components [34]. Medicinal herbs have a high moisture content after harvest, which fosters enzyme activity and microbial growth [35]. The efficiency of the secondary metabolites that give them their therapeutic qualities is diminished if the moisture content is not quickly decreased [36]. An inappropriate drying technique may damage the bioactive components in plant materials and degrade their quality. Reducing the water content of medicinal plants, which normally ranges from 75 to 80%, to less than 15% is essential for maintaining secondary chemicals and plant quality [24].

Drying is an essential step in the processing of medicinal plants to increase shelf life, preserve active chemicals, and facilitate transportation and storage [37]. It plays a critical role in the industrial processing and post-harvest management of these plants and is considered one of the simplest, most popular, and economical preservation methods. Selecting an appropriate drying method is vital for maintaining the quality and efficacy of various plant parts [38]. Medicinal herbs can be dried using diverse techniques, each with distinct advantages and limitations. Common drying methods include oven drying, sun drying, and shade drying. Modern techniques such as oven and microwave drying are recommended for preserving active compounds in plants with volatile and heat-sensitive constituents, while traditional methods like sun and shade drying are more suitable for plants less sensitive to light and heat exposure [39, 40].

The choice of drying technique depends on factors such as plant type, target chemical compounds, and desired processing outcomes. Despite being cost-effective and environmentally friendly, conventional methods like sun drying present several drawbacks, including time-consuming procedures, dependence on weather conditions, difficulty controlling drying rates, uneven drying, susceptibility to microbial contamination due to prolonged drying, exposure to environmental pollutants, and the need for large drying spaces [20, 40] Shade drying addresses the issue of direct sunlight exposure and often results in higher product quality compared to sun drying. However, it remains weather-dependent, and in cases of increased humidity or unexpected rainfall, rehydration of dried materials can occur [41].

Both sun and shade drying operate at low temperatures, which may help preserve heat-sensitive constituents in herbs. Nevertheless, their slow drying rates allow post-harvest metabolic activity to persist, potentially leading to the degradation of valuable chemical components [42]. product durability and ensure food safety, they often adversely affect bioactive ingredients, diminishing the nutritional and functional quality of the final product [43]. During drying, plant tissues lose moisture along with several volatile compounds [44]. Elevated temperatures further disrupt cell walls and plasma membranes, which may compromise membrane permeability [45].

Heat-sensitive phytochemicals, such as flavonoids and phenolics, are prone to rapid degradation and loss of antioxidant properties at high temperatures [46, 47]. The main mechanisms behind these degradations include volatilization, oxidation, and structural breakdown, with processes like hydroperoxide formation and subsequent decomposition into inactive by-products playing significant roles [48]. Shade drying is noteworthy for its superior ability to preserve bioactive compounds, as it reduces the degradation rate of thermolabile substances like flavonoids, TPC, and essential oils. However, the extended duration of shade drying increases the risk of mold growth if the drying process is not sufficiently expedited, which can lead to quality deterioration [37].

In contrast, sun drying offers a quicker and more cost-effective method but exposes plants to UV radiation and elevated temperatures, leading to potential degradation of bioactive compounds. For example, high temperatures can cause structural degradation of essential oils [49]. Moreover, in high-humidity environments, prolonged drying times may adversely affect plant quality [40]. Shade drying is particularly advantageous in regions with intense UV radiation, as it better maintains the color, aroma, and structural integrity of plant materials [50].

The analysis of how various drying techniques affect the overall amount of TPC and TFC in *A. rosea* reveals that each technique has a unique impact on how well these compounds are preserved. Due to extended exposure to direct sunshine, the data demonstrate that sun drying considerably lowers the overall TPC and TFC. Although it takes longer to dry, shade drying maintains these components better because it offers more protection against photodegradation and heat damage. The effects of oven and microwave drying also differ. According to Athira et al. [51], high-temperature oven drying can accelerate the degradation of heat-sensitive bioactive compounds, which consequently leads to a significant reduction in the measured TFC and TPC values, mainly due to thermal degradation and prolonged oxygen exposure.

In a study on *Solidago virgaurea*, Parsafar et al. [52] found that samples that were shade-dried had the highest TPC (30 mg GAE/g DW), while those that were oven-dried at 40 °C had the highest TFC concentration (7.95 mg RE/g DW). Additionally, the number of active metabolites decreased as oven temperature and microwave power increased. Similarly, *Arnica chamissonis* flowers that were shade-dried had the highest TPC (38.42 mg GAE/g DW), followed by flowers that were oven-dried at 40 °C (33.61 mg GAE/g DW), according to Asadi et al. [53]. Further supporting the beneficial impact of controlled drying conditions on the preservation of bioactive compounds, these findings are consistent with those reported by Mumivand et al. [54] or *Pelargonium graveolens*, with total phenol content of 14.78 mg GA per 100 g dry matter and total flavonoid content of 12.83 mg quercetin per 100 g dry matter.

Numerous bioactive substances, such as flavonoids, alkaloids, tannins, and saponins, are found in moringa leaves. These substances exhibit a range of pharmacological actions. Moringa leaves are the most prevalent source of flavonoids, which are quantified by the Total Flavonoid Content (TFC) assay. Kaempferol and quercetin are the two primary flavonoid compounds found in moringa leaves [55]. The antioxidant potential of this plant may be attributed to its TPC and TFC levels, lycopene, carotenoids, ascorbic acid, and anthocyanins [56]. Additionally, this plant’s TFC and bisphenols have shown anti-ulcer and gastroprotective properties [57, 58]. Antioxidants can prevent free radical molecules from oxidizing biological components, including proteins, membranes, and nucleic acids. Antioxidant-rich herbs can increase cellular resistance to free radical damage, reducing the chance of contracting diseases [59]. The effects of different drying techniques (both conventional and combination processes) on the antioxidant properties of herbs have thus been extensively studied. The primary source of the antioxidant qualities of medicinal plants and the products made from them is the presence of phenolic antioxidants from different classes. Oxygenated monoterpenes are phenolic antioxidants because they include reactive phenolic fragments. The majority of terpenes are found in medicinal plants [60].

Acetic acid has been shown to reduce oxidative stress by fortifying plants’ antioxidant defense system by increasing both enzymatic and non-enzymatic antioxidant levels, in addition to improving the photosynthetic potential of a range of plants. It has been observed that applying acetic acid to soybean plants increases the activity of important antioxidant enzymes, reducing oxidative stress that is caused [61]. Similar findings have been documented in a range of stressed plants regarding the acetic acid-mediated suppression of ROS as a result of the antioxidant defense system being strengthened [62].

There are sterols, TFC, terpenes, and alkaloids in *Moringa oleifera* [57]. These metabolites have shown antidepressant and anxiolytic effects in several animal models of anxiety by stimulating the noradrenergic, serotonergic, and dopaminergic neurotransmitter systems and interacting with the GABA subtype A receptors (GABAA-receptors) [63]. Furthermore, these compounds produce their anxiolytic effects by acting as either agonists of benzodiazepines and GABA on the benzodiazepine/GABA receptors or antagonists of N-Methyl-D-Aspartate (NMDA) on the NMDA receptors [64].

Glutathione (GSH) and catalase (CAT) levels were markedly elevated after administering *Moringa oleifera*. It has been demonstrated that GSH, a potent antioxidant, guards against damage from free radicals. It is present in practically all mammalian tissues and is composed of three amino acids: glutamic acid, cysteine, and glycine. GSH is a detoxifying and antioxidant molecule that scavenges free radicals [65].

Numerous plants, animals, and microorganisms contain the four-carbon nonprotein amino acid GABA. GABA is found in practically all advanced plants. Plant stress tolerance is correlated with GABA. Following environmental stressors like high salt, low temperature, oxidative stress, etc., plants’ GABA content will rise noticeably [66]. Numerous plant species have been reported to contain GABA and glutathione. The best foods to get these amino acids include vegetables (spinach, broccoli, onion, potatoes, sweet potatoes), fruits (squash, apples, blueberries), cereals (brown rice germ, brown rice sprouts, barley sprouts, bean sprouts, beans, corn, barley, and brown rice), and some herbs (chamomile, salvia, lavender). There is little information currently available regarding GABA’s existence in therapeutic plants [67, 68]. The richest source of GABA was found in the roots of the herb bistort (*Polygonal bistort* L.), and this is the first report on its GABA content. GABA is also abundant in basil, lophantus, and chamomile flowers. Interestingly, it has been demonstrated that chamomile, basil, and white oregano all have anxiolytic qualities. For chamomile, this effect was attributed to TFC (apigenin) [69], whereas for the other herbs, it was attributed to certain phenolic compounds from the essential oil [70]. Natural products, especially some phenolic substances, are known to modulate GABA receptors [71].

Glycine is a neutral, nonpolar amino acid that is produced during photorespiration from serine and is one of the 20 necessary amino acids for plants. A portion of glycine can be transformed into glutamate, which can subsequently undergo metabolism to produce GABA [72]. It is already established that GABA has a crucial role in controlling plant growth and development, moderating the response to stress tolerance brought on by salt, low light, nitrogen insufficiency, water deficit, and high temperatures, and controlling antioxidant defense systems [73]. Glutamate is recognized as a key signal for biotic and abiotic stress tolerance through glutamate receptors (GLRs) [74]. Two other plant pathways may be connected to glycine’s impact on stress tolerance, albeit they haven’t been confirmed yet. First, the well-known protection against plant stressors provided by glycine betaine (GB) [75]. Choline, which comes from the same metabolism as glycine, is used to make GB [76]. Additionally, glycine’s activation of GLRs can boost antioxidant metabolism, which in turn can lessen plant stress [77]. Furthermore, glycine contributes to the construction of a number of proteins, primarily those involved in the creation of the cell wall, of which glycine forms around 70% [78].

## Conclusion

This study showed that different drying methods significantly affect the content of active compounds and the quality of *Moringa oleifera*. Sun and oven drying are suitable methods for preserving TPC, TFC, salicin, and glycine, while shade drying is recommended for preserving antioxidant activity, acetic acid, butyric acid, and GABA. These findings suggest that selecting the appropriate drying method, tailored to the desired active compounds, is crucial for maintaining the quality and efficacy of Moringa. For pharmaceutical applications where the preservation of TPC, TFC is important, sun or oven drying is suitable. For applications where high antioxidant activity is desired, shade drying is recommended. Finally, this study emphasizes the importance of optimizing drying methods to preserve the bioactive compounds of medicinal plants and ensure their efficacy in various applications. Further research into the impact of combined and more advanced drying methods could help develop more efficient and effective methods for preserving the quality of medicinal plants.

## Data Availability

“All relevant data are included in the manuscript.”
